# Investigating a shared-dialect effect between raters and candidates in English speaking tests

**DOI:** 10.3389/fpsyg.2023.1143031

**Published:** 2023-03-30

**Authors:** Ying Xu, Mengjia Huang, Jin Chen, Yaqing Zhang

**Affiliations:** School of Foreign Languages, South China University of Technology, Guangzhou, China

**Keywords:** English speaking tests, shared-dialect effect, accent, stimulated recall, retelling

## Abstract

This study set out to examine existence of a shared-dialect effect, a phenomenon that when a rater shares the same dialect with a candidate, the rater is more likely to give the candidate a higher score in English speaking tests. Ten Cantonese-speaking raters and ten Mandarin-speaking raters were selected to assess forty Cantonese-accented and forty Mandarin-accented candidates’ oral performance in the retelling task of the Computer-based English Listening and Speaking Test (CELST). Besides, seven raters from each group participated in the stimulated recall stage aiming to reveal their thought process. Quantitative results suggested that the two rater groups were comparable in terms of internal consistency. There were no significant differences in the scores of both candidate groups awarded by both rater groups. The effect of interaction between candidates’ dialect and raters’ dialect was not statistically significant, indicating non-existence of such effect. Qualitative results showed that some raters attended to candidates’ accents, and indicated that awareness of accents and their familiarity with the accents affected their comprehension of the speech samples and potentially influenced their scoring process. The findings are discussed with reference to rater training, rating scale, raters’ familiarity with candidates’ accents, raters’ attitudes toward candidates’ accents and the task type. The main implication of this study is that recruiting both group raters in domestic English speaking tests is warranted if the shared-dialect effect could be duly managed.

## Introduction

Raters’ judgment plays an indispensable part in oral performance assessments, which may be easily affected by construct-irrelevant factors (e.g., rater bias) and bring a detrimental effect on test fairness. However, there is a possibility that raters are affected by various background factors that are not involved in the rating criteria during the rating process, and those factors could introduce unwanted, construct-irrelevant variance into ratings, thus interfering with the measurement of speakers’ actual performance and unrightfully contributing to the score variance (McNamara, 1996; Winke, 2012). Those construct-irrelevant variations in scores such as bias against non-native accents (Lindemann, 2017), also termed rater effects, must therefore be minimized to avoid unduly influencing raters’ scoring decisions, especially in large-scale and high-stakes speaking assessments (Winke et al., 2013; Isaacs, 2016; Kang et al., 2019a). Given the importance of validity and reliability in assessment design and development, raters are supposed to refrain from any performance-irrelevant judgments and research on the impact of rater background characteristics on their ratings have captivated many researchers’ attention, as the understanding of raters’ rating behaviors is conducive to elucidating “why raters assign ratings the way they do and what attributes or elements they still need to improve their rating performance” (Kim, 2015, p. 241). A large volume of research has been carried out in an attempt to investigate the influence of various rater background characteristics on raters’ rating behaviors and cognitive process in speaking assessments, such as raters’ linguistic background (Zhang and Elder, 2011; Gui, 2012; Wei and Llosa, 2015), rater experience (Isaacs and Thompson, 2013; Kim, 2015) and rater training (Weigle, 1998; Xi and Mollaun, 2009; Davis, 2015), but no definite conclusion can be reached due to the complex nature of raters’ decision-making process. Among the listener background variables, familiarity with the accent has increasingly garnered research interest because listeners are likely to be confronted with a range of native speaker accents and diverse non-native accents in various language use contexts (Canagarajah, 2006). A considerable amount of literature in the field of speech processing and language assessment has investigated the impact of accent familiarity on listeners’ perceptions and judgments (Xi and Mollaun, 2011; Winke et al., 2013; Huang et al., 2016; Park, 2020). Nonetheless, there have been few empirical investigations into the effect of accent familiarity with a certain dialect on raters.

China is a multi-ethnical country with numerous dialects. There are at least eight main dialects in China (Li, 1989), among which Cantonese and Mandarin are most widely used (Lee et al., 1996). Mandarin and Cantonese are tone languages belonging to the Sino-Tibetan language family (Yang et al., 2020). Mandarin is the official language of China with more than 1 billion speakers worldwide. Cantonese, as one of the most well-known Chinese dialects, is conservatively estimated to have over 60 million speakers in the world and is mainly spoken in Guangdong province, the Southeast region of Guangxi Province, Hongkong, and Macau (Han et al., 2015). In the field of applied linguistics, Mandarin and Cantonese are generally treated as two distinct dialects for their disparate phonological systems. For example, in Munro et al.’s (2006) study, Cantonese was treated as the phonetic counterpart of Mandarin. A more radical view even deems them as two languages with representative phonetic features which can reach the international standard of bilinguals (Xing et al., 2021). Some apparent phonological discrepancies were found between Mandarin and Cantonese in terms of tones, vowel and consonant sounds, as well as accents, evidenced by the fact that there are five tones in Mandarin whereas Cantonese has practically nine tones. When it comes to the phonetic symbol system, the Mandarin vowel system comprises nine monophthongs, nine diphthongs, and four triphthongs, while Cantonese has 11 monophthongs and 11 diphthongs in its vowel system and 19 initial consonants, and six final consonants in its consonant system (Law and So, 2006). Cantonese-accented English is characterized by some typical pronunciation errors, such as sounds’ swallowing (/d/,/t/,/k/,/z/), sounds’ addition, phoneme error, word or chunk error and word stress errors (Xu and Zeng, 2015). Nevertheless, the following acoustic properties such as the devoicing of a word-final stop consonant (Hayes-Harb et al., 2008), the mispronunciation of/ae/,/*ε*/,/∧/, and the improperly perceptual distance of tense and lax vowels (Barkana and Patel, 2020) are labeled Mandarin-accented English. Winke et al. (2013) claimed that Cantonese and Mandarin as different language varieties might affect raters’ judgment toward speakers’ oral performance. Therefore, of interest to this study is whether raters assign scores to examinees’ performance differentially as a result of different dialects spoken by the rater and the candidate.

In 2011, the National Matriculation English Test (NMET) of Guangdong province incorporated a separate component of oral test, the Computerized English Listening and Speaking Test (CELST), which purports to gauge candidates’ oral English ability. The annual number of candidates for the CELST in Guangdong NMET amounts to over 800,000 and they are from all over the province (Zhang, 2014). Speech produced by these candidates would inevitably be stamped with their native dialects, and human raters invited to assess candidates’ oral performance may also be affected by the dialects they routinely speak. Thus, there is an urgent need to scrutinize whether there exists a shared-dialect effect, analogous to a shared-L1 effect (Harding, 2012), between raters and examinees in oral English proficiency tests, and whether raters are aware of any influences of such effect on their scoring.

To the best of the authors’ knowledge, any research that has specifically explored whether raters’ background difference in dialects could cause noticeable differences in their ratings or make a difference to their decision-making process in English speaking tests is yet to be carried out in the Chinese context, which is demonstrably a crucial question requiring an immediate answer. Therefore, it is urgent to inquire existence of a shared-dialect effect and to elucidate whether certain candidates are thus advantaged or disadvantaged. The research results could advance our knowledge of such effect and shed light on the recruitment of raters with regional dialects in English speaking tests.

## Literature review

### Empirical studies on the shared-L1 effect

In the field of language testing, the term shared-L1 effect refers to the phenomenon that a group of candidates who share the same L1 with the speaker of the test recording can find the listening materials more comprehensible and give a better performance on the test (Harding, 2012; Dai and Roever, 2019), which is analogous to the *interlanguage speech intelligibility benefit* (ISIB) in the speech processing literature (Bent and Bradlow, 2003). A probable explanation for this phenomenon might be that being exposed to a speaker’s accent repeatedly contributes to familiarity with that accent, which in turn facilitates comprehension of the speaker (Bradlow and Bent, 2008; Stevenage et al., 2012). Although the possibility of a shared-L1 advantage and the potential for a shared-L1 effect have aroused considerable interest in areas including speech perception, L2 listening comprehension and language testing, current research has yielded mixed results.

The term ISIB refers to the benefit of a shared language background between non-native listeners and speakers. It was proved by Bent and Bradlow (2003) which performed perception tests on native speakers of Chinese, Korean, English, and other language backgrounds, asking them to listen to sentences read in English with Chinese, Korean, and English accents. Results indicated that native English listeners had higher word recognition rates for sentences spoken by native than non-native speakers. However, the non-native listeners found high-proficient non-native speakers of the same L1 equally as intelligible as the native English speakers. Interestingly, there seems to be an assumption that the L2 proficiency of listeners and speakers may play a role in modulating the ISIB (Xie and Fowler, 2013). A few studies suggested that the interlanguage speech intelligibility benefit existed more in low-proficiency learners. For example, in a follow-up study of Bent and Bradlow (2003), Stibbard and Lee (2006) reported that there were no significant differences in intelligibility scores for high proficiency non-native speakers and native speakers within each listener group. Native speakers were not more intelligible than non-native speakers even to their fellow native listeners. The non-native listeners found that the non-native low-proficiency speakers who shared their own first language was not as significantly unintelligible as those who did not share the first language, which suggested that the shared-L1 effect may only be taking hold when listeners heard lower-proficiency speakers.

Additionally, from the perspective of language features, the sheer volume of studies provides partial or little evidence to support a shared-L1 advantage phenomenon. Some studies such as Harding (2012), Dai and Roever (2019) found positive evidence of the shared-L1 effect in Mandarin-L1 candidate groups through the comparison of candidates’ performance in English listening tests conducted with various accents. The Mandarin Chinese-L1 listeners were found distinctly advantaged on several test items featuring a speaker with Mandarin Chinese accent. However, studies conducted by Major et al. (2002), Kang et al. (2019b) failed to support the shared-L1 effect argument for the Chinese-L1 listeners scored significantly lower than other listener groups when listening to passages recorded by speakers who shared their native language. Other studies, such as Munro et al. (2006) have shown the facilitative effect of L1 accent on the Japanese listeners group on account of the fact that researchers found speeches produced by speakers of their own language background were easier to understand than speeches by Cantonese, Polish and Spanish speakers. Nonetheless, in Harding’s (2012) study, the effect of shared-L1 was not clearly observed when investigating a shared-L1 advantage to the Japanese. The mixed findings grounded in the above two languages could not offer full support for the existence of the shared-L1 effect. Regarding other languages that have been investigated, Abeywickrama (2013) found no evidence of a shared-L1 effect by the measurement of three other language groups’ (Korea, Sri Lanka, and Brazil) comprehension of shared-L1 accent speech *via* a multiple-choice (MC) TOEFL listening test whose speech stimulus were recorded by speakers with Chinese, Korean, Sri Lankan, and American accent. Test-takers’ comprehension scores on the MC listening assessment were not significantly affected by speakers’ accents and they had comparable performance even when the input was delivered by speakers who shared the same native languages, suggesting that there is no shared-L1 effect. Besides, the shared-L1 advantage has not yet been found in the French-language background. For example, Crowther et al. (2016) examined how listeners’ status (native, non-native) and language background (French) influenced the raters’ (French, Mandarin) L2 comprehensibility and accentedness. Analyses of the global ratings demonstrated that when rating the L2 speakers from the French-language background, the French listener group did not benefit from the shared language background compared to the Mandarin listener group, contradicting the shared-L1 advantage.

These mixed findings reported on various languages have shown the indeterminacy of the existence of the shared-L1 effect, which suggests that the effect is not consistent across language variables. The question of whether shared-L1 could impact candidates’ performance in listening tests still remains unknown. More importantly, prior studies predominantly focused on the language of a certain country without the consideration of its regional varieties’ effect on research findings (Winke et al., 2013). In China, Mandarin Chinese speakers might put on diverse local accents across the country. For fairness reasons and positive washback of language tests, there is a necessity to examine the effect of Chinese dialects on the interactions between listeners and speakers under Chinese dialect cultural contexts.

### The accent familiarity’s effect on raters

Familiarity with a particular accent is conducive to understanding that type of accented speech (Gass and Varonis, 1984; Tauroza and Luk, 1997; Major et al., 2002; Dai and Roever, 2019). To date, several studies have examined the influence of accent familiarity of certain languages on raters’ rating process and behaviors (Carey et al., 2011; Huang, 2013; Winke et al., 2013; Park, 2020). Results of following studies suggested that raters’ familiarity with examinees’ accents affects the rating of pronunciation and general speaking ability. For example, Carey et al. (2011) demonstrated that raters who were familiar with the candidates’ accent were more likely to assign favorable higher pronunciation scores than raters who had little or no familiarity with that accent, and they also tended to score candidates from their own home country higher than candidates from a different country. Their findings were similar to Winke and Gass (2013) which delved into raters’ cognitive process through collecting raters’ (Spanish, Chinese, and Korean L2 learners) comments while rating three groups of examinees from Spanish, Chinese, or Korean L1 backgrounds in a qualitative study. Analyses of raters’ comments revealed that heritage language speakers had unconscious biases in rating familiar accented speech samples. This result supported the notion that raters’ language backgrounds, in particular heritage language backgrounds, could influence their rating decisions. It demonstrated the potential bias of accent familiarity on raters’ scoring and also provided evidence supporting Winke et al.’s (2013) hypothesis that accent familiarity could potentially lead to bias, including rating reliability, though the effect may be limited and inconsistent. However, such a clear pattern was not observed by Park (2020) which found that ratings across three teacher groups with different degrees of familiarity with Korean accent (heritage, familiar, and unfamiliar) on the assessment of Korean-accented English exhibited high interrater reliability, and prior exposure to foreign-accented speech affected their consistency in ratings. In the meanwhile, by comparing the severity of the three groups, the researcher found that non-heritage teachers were less tolerant than heritage teachers in assessing global proficiency and accentedness, even though there was no significant difference in the level of severity between the familiar and unfamiliar teacher groups.

The causes of inconsistent results are likely in part due to different methodological perspectives, the tools used to measure familiarity, and raters’ varying perceptions of interlingual and intralingual accents. It should be noted that the first three studies in this section examined raters’ familiarity effect on the assessment of L2 pronunciation by comparing rater performance while assessing speech samples with different accents, but these studies did not strictly control raters’ familiarity with every accent. Although Park (2020) investigated the familiarity’s effect with a simple accent from different levels, the current literature is still limited and further research should give clear evidence to illustrate the familiarity effect on rater bias.

### Raters’ perception of candidates’ accent

Contrary to the significant effect of accent familiarity manifested in the reviewed studies, other studies failed to detect that effect (Xi and Mollaun, 2009, 2011; Huang, 2013; Wei and Llosa, 2015; Huang et al., 2016) under various conditions. However, it does not mean that raters’ decision-making would not be affected by other mediating variables. It’s still necessary to further explore the potential effect of raters’ complicated psychological course ensconced in digital signals transmitted by scores. The deep-going comportment can reveal the possible factors that would lead to raters’ differential assessment decisions with different accents and provide insights into raters’ views of the practicality of including non-native accents in English speaking tests.

Xi and Mollaun (2009, 2011) compared the ratings of the TOEFL iBT Speaking test assigned by trained (including how to score English speech samples from native-Indian speakers) and untrained bilingual/multilingual Indian raters. Even though they did not find a significant difference between the numerical ratings, they discovered positive effects from undergoing the training, which helped trained raters guard against what they claimed to be an internal dilemma when rating speakers of familiar accents. Huang’s (2013) findings were consistent with those of Xi and Mollaun (2009, 2011), showing no significant differences between the ratings of the three rater groups. With a focus on raters sharing the same L1, Huang (2013) from the angle of teaching experience as well as accent familiarity, investigated the two-fold effect on raters’ self-perception. Three groups of raters who varied on familiarity with non-native accents and language teaching experience were recruited to evaluate speech samples spoken by native Chinese speakers on both holistic and analytical dimensions. Results revealed that the speakers’ accent together with teaching experience might lead to the potential leniency effect. Given that raters’ bias related to the two factors’ combining effect, it was unclear whether accent familiarity alone could give rise to rating bias. In the subsequent study of Huang et al. (2016), they only investigated the influence of raters’ familiarity with accents on their rating decisions. Three groups of raters with different backgrounds (Spanish Heritage, Spanish Non-Heritage, and Chinese Heritage) rated 28 speech samples on the overall English proficiency and foreign accents. Raters self-reported that their accent familiarity affected their evaluations of accentedness, and might have made them more lenient toward speakers with familiar accents. Besides, they expressed a strong preference for Spanish accents. Results clearly demonstrated that being familiar with a certain type of foreign accent facilitated the identification of that accent and also revealed that more favorable accents in their study were those prevalent in the language speaking country, suggesting that positive contexts of familiarity would lead to positive bias and vice versa (Cargile, 1997; Lindemann, 2005). Similar findings from the quantitative view were also obtained in Wei and Llosa (2015), which examined whether American and Indian raters differed in their scores and scoring process with Indian test-takers’ speech samples from the TOEFL iBT speaking tasks. No statistically significant differences were found between Indian and American raters in their use of the scoring criteria, their attitudes toward Indian English, and the internal consistency and severity of the scores. However, in-depth qualitative analysis revealed that some Indian raters even held negative attitudes toward Indian English. The findings of this study manifested that sharing a common language background does not guarantee a positive evaluation of candidates’ L2 speaking performance after all.

The inconsistent findings of quantitative and qualitative methods are unsurprising because of the complex development trajectories of cognitive processing in raters from various backgrounds. This mismatch also indicated that rater bias was not fully uncovered in reviewed studies or, alternatively, raters’ mental process was not precisely captured for the methodological gap.

In summary, the aforementioned studies have produced somewhat inconclusive results regarding the shared-L1 effect based on different language backgrounds of listeners and speakers, and existence or strength of such effect has not been fully investigated. Scant literature has been found focusing on the impact of sharing the same dialect between raters and candidates in English speaking tests, let alone the potentiality for a shared-dialect effect in the Chinese context. Although the role of raters’ accent familiarity of certain languages in speaking assessments has received increasing attention, more empirical research is needed to further probe the effect of accent familiarity of dialects subsumed under one certain language on rating performance and cognition.

### Research questions

The overarching goal of this study was to explore the potential for a shared-dialect effect in English speaking tests in the Chinese context and to investigate whether raters were aware that the shared dialect between raters and candidates may have an influence on their judgment of oral performance. The present study was guided by the following two research questions:

(1) Are there any significant differences in the scores given by Cantonese-speaking and Mandarin-speaking rater groups to the Mandarin candidate group and the Cantonese candidate group on the Retelling task in the CELST?
(2) Are trained raters aware that the shared dialect with the candidates might impact their ratings on the Retelling task in the CELST?

## Methods

### Participants

Thirty-eight postgraduates from different universities in China were recruited as raters. Graduate students were selected because participants from a more diverse population would introduce far more variables (Winke et al., 2013). One half of them were heritage speakers of Mandarin and the other half Cantonese heritage speakers, meaning that they were immersed in the language environment where their family members spoke that language natively and were identified with one particular ethnic group by it. They were all female aging from 22 to 26, none of whom had hearing or speech disorders. All of them had been learning English as a foreign language in China for at least 12 years. Following the six steps training approach proposed by Bachman and Palmer (1996, p. 222) and the calibration standard suggested by Hoskens and Wilson (2001), only 20 raters were accredited and they were classified into two background groups according to their dialects: Group A (including ten Mandarin-speaking raters) and Group B (including ten Cantonese-speaking raters). No participant reported speaking any languages or dialects other than Cantonese, Mandarin and English.

To avoid any influence caused by background variables, the participants were selected on the basis of homogeneity of their educational background, language proficiency and rating experience. First, all raters were postgraduate students studying in the field of applied linguistics. Second, they had all passed Test for English Majors for Grade 8 (TEM8) (Jin and Fan, 2011). TEM8 is a large scale and high-stakes criterion-referenced English test, designed to assess undergraduate English majors’ language proficiency at the end of their four years professional learning program (Zou and Xu, 2016), to check whether test-takers’ language knowledge and capacities could meet the learning requirements documented in the *Syllabus for Test for English Majors (Grade 8)* (National Advisory Committee for Foreign Language Teaching, 2004). Last, they all had no prior rating experience of any oral assessments. They were informed that they should attend both the training stage and the rating stage. Besides, 14 raters (seven raters from each group) were invited to take part in the stimulated recall stage based on their availability. All participants received certain monetary rewards for their participation. Raters’ general background information was collected with an online background questionnaire before training, which would be introduced in the forthcoming section. Some detailed background information of raters is shown in Table 1.

**TABLE 1:** Raters’ background information.

Group	Dialect	Rater	Age	Years of learning English	Familiarity with Cantonese	Attitude toward Cantonese
A	Mandarin	A1	23	14	2	5
		A2	25	13	2	3
		A3	24	15	1	4
		A4	24	14	2	4
		A5	24	13	2	4
		A6	25	14	1	3
		A7	25	16	2	4
		A8	26	16	2	4
		A9	23	15	1	5
		A10	22	12	2	4
B	Cantonese	B1	24	15	5	5
		B2	25	16	5	5
		B3	26	14	5	4
		B4	24	15	4	5
		B5	24	14	5	4
		B6	23	13	5	5
		B7	23	13	4	5
		B8	24	15	5	5
		B9	26	16	5	5
		B10	25	14	4	4

The referential meanings of the last two columns’ numbers are further elucidated in the background questionnaire section.

An independent *t*-test performed on the familiarity with Cantonese showed that there was a significant difference between the two groups (*t* = −13.887, df = 18, *p* = *0.00*). No significant statistical difference was found in the means of age (*t* = −0.590, df = 18, *p* = 0.56) and years of learning English (*t* = −0.557, df = 18, *p* = 0.58). Only two Mandarin-speaking raters reported that they were neutral about Cantonese, but the rest of the raters held a positive attitude toward Cantonese.

### Instruments

#### Speech samples

The speech samples for the present study were candidates’ performances on the Retelling task in the CELST in Guangdong NMET in 2013 (Appendix A). Four subsets of samples in different numbers were purposefully chosen from a pool of sound files by 32 listener judges who were enrolled in a MA programs at a University in Guangdong, with either Cantonese or Mandarin background. In order to strictly control all the speech samples to have a similar degree of accent strength and identifiability, 32 recruited listener judges were required to evaluate the above two mentioned indexes of the provided speech samples with a Strength and Identifiability of Accent Scale (Appendix B), which was designed drawing on Ockey and French’s (2016) accent scale and the accent strength and identification task used in Dai and Roever (2019). To guarantee reliable accent strength and typicality, only judges who claimed high familiarity with the two dialects in the evaluation process and reported to be apt at dialect judgment and identification were selected. At last, 96 valid speech samples were included and classified into four subsets in the formal experiment.

Subset 1 included four benchmark samples, used as exemplars of each score band of the rating scale, representing a range of proficiency levels and performance types. Subset 2 contained 12 practice samples rated by two expert raters (who were professors of applied linguistics and had more than eight years rating experience of CELST) and used in training. Subset 3 consisted of 80 formal rating samples utilized in the formal rating. Subset 4 comprised four Cantonese-accented speech samples and four Mandarin-accented speech samples, which were purposefully picked out from Subset 3 and used as the prompts in the stimulated recall stage. All raters rated and commented on the same set of speech samples in the experiment. The formal rating samples were counterbalanced in terms of candidates’ dialect (Mandarin, Cantonese), gender, and official NMET scores of the Retelling task. Candidates were evenly divided into two groups based on their dialects. Each dialect group had 20 male and 20 female candidates. Ten candidates (five males and five females) were at each of two levels of proficiency (high and low) within each dialect group. The two levels of proficiency were assigned according to candidates’ NMET scores of the retelling task. As the maximum score of the task is 24, candidates who received a score higher than 18 were labeled high-level, and those scored lower than 12 low-level. Samples with the same dialect, of the same gender and of the same proficiency did not occur adjacently.

#### Background questionnaire

At the beginning of the study, all participants completed a background questionnaire online (Appendix C) to obtain participants’ demographic information and to explore their language background. By adapting the questionnaire from Wei and Llosa (2015), questions in this instrument aim to solicit information concerning raters’ age, gender, dialect, English learning experience and proficiency levels, exposure to Cantonese, rating experience and academic background. In addition, participants’ familiarity with Cantonese was also gauged on a Likert-scale ranging from 1 (strongly unfamiliar) to 5 (strongly familiar) after listening to two pieces of speech materials with typical pronunciation characteristics of the Cantonese accent. Similarly, a 1–5 Likert scale (1 = strongly dislike; 5 = strongly like) was employed to investigate participants’ attitudes toward Cantonese. The reason why not tap into raters’ familiarity with and attitudes toward Mandarin is that it is the official national language and has been popularized in China for several decades, hence generally Chinese people are much familiar with it and hold a positive view on it.

#### The retelling task

The retelling task in the CELST is designed to measure candidates’ integrated listening and speaking ability, especially the ability to obtain information from listening materials and to process and reconstruct information. Candidates are required to listen to a 2 min story. While listening, they are presented with a one-sentence hint of the story. The story will be played twice. Candidates are allowed to take notes while listening. After listening and 1 min preparation, they should retell the story by using proper words and sentences within 1 min. The retelling content should cover as much information of the story as possible. The entire process of completing a retelling task in the CELST lasts approximately 6 min.

The present study only concentrated on the retelling task because as a typical integrated task, retelling could reflect candidates’ use of second language in the real-life situations and measure candidates’ speaking ability validly, thus has been widely used in L2 oral performance assessments (Frost et al., 2012).

#### Rating criteria

Rather than using the official rating scale of retelling in the Guangdong NMET (Appendix D), the modified version of the rating scale of TEM4 (Test for English Majors for Grade 4) story retelling task developed by Liu (2013) (Appendix E) was employed, because the official rating scale (including two dimensions: Content and Holistic) does not require raters provide any score on candidates’ pronunciation, which is a major concern of the present study. Instead, Liu’s (2013) version is an analytic rating scale, containing four conceptual dimensions: Grammar, vocabulary and expression; Retelling content; Pronunciation and intonation; and Fluency. There are detailed descriptions of four different levels in each dimension. The full mark is 16 points because each dimension spans score bands of 1 (lowest) to 4 (highest) corresponding to the different levels.

#### Stimulated recall

Stimulated recall was conducted to trace raters’ individual thinking process in assigning scores. As an introspective method, this type of verbal reporting is conducive to probing into the complex nature of the scoring process by providing raters with recently recorded stimulus or cues (Gass and Mackey, 2000). It is generally applied in studies of rater performance in speaking tests (Winke et al., 2013). By replaying the tape-recording or fragments of the recording, stimulated recall could prompt raters to recall and verbalize their concurrent cognitive activity when performing the scoring task.

### Procedures

**FIGURE 1:**
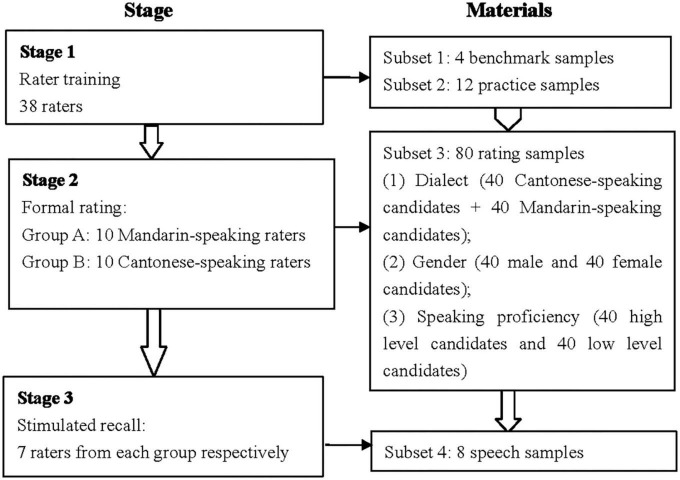
The procedure.

This study included three stages: training, rating, and stimulated recall. Due to the outbreak of *COVID-19* pandemic, training, and stimulated recall were carried out on *Tencent Meeting*, an application allowing users to attend a real-time interactive online meeting. Figure 1 illustrates the details.

Before starting the actual rating, each participant should undertake training. At this stage, 38 raters were provided with the original script and the recording file of the story-retelling, the rating criteria, benchmark samples, and practice samples. Firstly, all raters were allocated enough time to familiarize themselves with the task and the rating criteria. Secondly, one researcher introduced the story-retelling task, and then illuminated the rating criteria in detail and explained the rationale for assigning a specific score for speech samples at each score level with the benchmark samples. Raters could ask any questions concerning the rating scale in order to internalize it with the help of benchmark samples. Next, each rater was asked to rate the set of 12 practice samples and provide legitimate reasons for their ratings individually. Later, they compared their rating results and reasons with the scores and rationales provided by the two expert raters. In an attempt to simultaneously guarantee the reliability and validity of the formal rating, only 20 raters who not only reached 80% consensus with the agreed-upon scoring outcomes but also correctly interpreted the rating scale reflected by their reasons for ratings were accredited (Hoskens and Wilson, 2001; Elder et al., 2007; Xi and Mollaun, 2009). Last, seven raters in each group were informed of the procedure for stimulated recall and trained to verbalize their thoughts. After practicing with a sample recording, no participant reported difficulty in verbal reporting.

At the formal rating stage, raters were allowed to complete rating independently with the rating scales at their convenience within a certain time on the computer. They were allowed to listen to the speech samples for more than one time if necessary.

When each of 14 selected raters completed the rating tasks, she was arranged to converse immediately with one researcher to undergo the stimulated recall stage individually. At this stage, the recordings of four Cantonese-accented and four Mandarin-accented speech samples were replayed twice to raters by the researcher. They should award a score to the current candidate’s performance in the same way of the rating stage and state the reasons for that score after the first replay. Then after listening for a second time, they were encouraged to recall what they had been thinking about at the time of rating and speak out what came to their minds immediately as much as possible. Leading questions were shown below: (1) What were you thinking about when scoring? (2) What were you thinking when listening to the speech sample? (3) Did you find this sample difficult to understand? Why? What factors affected your understanding? Any further ideas or comments if raters wished to elaborate were welcomed. Raters were free to choose any language to verbalize their thoughts, so that they could express their ideas fluently and clearly. The whole procedure was audio recorded, lasting for approximately 60 min.

### Data analysis

Quantitative and qualitative data have been gathered for the present study. Quantitative data consisted of 1,600 valid ratings that were assigned by 20 raters to 80 speech samples, and qualitative data included the voice recordings of the stimulated recall. A combination of quantitative and qualitative methods was used in the data analysis.

To answer RQ 1, raw data was collected by using Microsoft Excel spreadsheets. The interrater reliability statistics were calculated at first and the Two-Way factorial analysis of variance (ANOVA) was performed to examine whether there were any significant differences in the scores of two candidate groups awarded by two rater groups through the software SPSS 19.0.

To address RQ 2, an analytic inductive approach (Thomas, 2006) was adopted to analyze qualitative data collected through stimulated recall. Themes and patterns were expected to emerge from the data. Verbal reports were analyzed in four steps. Firstly, all stimulated recall audio recordings were transcribed verbatim by a research assistant majoring in language testing and double-checked by one researcher. The essential principle of the transcription was faithfulness. In the transcribing process, the playing of recordings of speech samples was omitted and all spoken information on the recordings from the scoring and reporting sessions should be written down as much as possible. Any pause longer than 3 s was marked by a “…”. Then the transcribed texts of each rater were entered into the qualitative data analysis software QSR NVivo 11.0. Next, the research assistant segmented those transcripts into idea units (Green, 1998) independently, which were double-checked by the researcher. For the sake of coding reliability, the researcher and the research assistant reread all the idea units and coded them into various themes independently. Finally, they discussed and agreed on specific names for, and operationalization of prominent themes. Through discussion, a consensus was reached on coding.

## Results

### Findings of RQ 1

#### Rater reliability and descriptive statistics

An internal consistency was examined by means of reliability analysis. Results showed that Cronbach’s coefficient alpha was 0.961 and 0.988 for the scores awarded by Cantonese-speaking and Mandarin-speaking raters, respectively. The reliability statistics indicated that the two rater groups exhibited high internal consistency. Furthermore, descriptive statistics for 80 candidates’ scores assigned by the two rater groups were reported in Table 2.

**TABLE 2:** Two-factor ANOVA results for within group analysis.

	Cantonese-speaking rater group				Mandarin-speaking rater group					
**Dimension**	***N***	**M**	**SD**	**SE**	***N***	**M**	**SD**	**SE**	**F**	**Sig.**
Grammar, vocabulary and expression	10	2.81	0.72	0.08	10	2.78	0.69	0.08	0.099	0.75
Retelling content	10	2.87	0.85	0.09	10	2.69	0.82	0.09	1.314	0.17
Pronunciation and intonation	10	2.86	0.66	0.07	10	2.93	0.69	0.08	0.523	0.47
Fluency	10	2.86	0.70	0.08	10	2.90	0.68	0.07	0.089	0.77
Total score	10	11.40	2.83	0.32	10	11.30	2.79	0.31	0.059	0.81

*N*, number; M, mean scores; SD, standard deviations.

It can be seen that for the four dimensions and the total score, the means of scores given by the Cantonese-speaking raters were either slightly higher or lower than those by the Mandarin-speaking raters, yet no statistically significant difference was observed (*p* = 0.171∼0.809).

### ANOVA results

A mixed two-factor ANOVA with raters’ dialect (the between-subjects factor) and candidates’ dialect (the within-subjects factor) as independent variables and the total score as the dependent variable was initially run to test whether differences in ratings across the two groups were statistically meaningful. It was found that there was non-significant difference for candidates’ dialect [F (1, 1) = 2.078, df = 1, *p* = 0.15] and raters’ dialect [F (1, 1) = 0.059, df = 1, *p* = 0.81]. The interaction effect between raters’ dialect and candidates’ dialect [F (1, 1) = 0.000, df = 1, *p* = 0.99] was not statistically significant either.

In order to test whether differences in ratings along four dimensions across the two groups were meaningful, two-factor ANOVA was used four times. Due to multiple comparisons being made, a Bonferroni correction was applied to the *p*-value with a new threshold of 0.0125 set.

First, using scores in *Grammar, vocabulary, and expression* as the dependent variable, it was found that there was no statistically significant main effect of candidates’ dialect [F (1, 1) = 2.397, df = 1, *p* = 0.12], no statistically significant main effect of raters’ dialect [F (1, 1) = 0.099, df = 1, *p* = 0.75], and no statistically significant interaction effect between candidates’ dialect and raters’ dialect [F (1, 1) = 0.013, df = 1, *p* = 0.91]. Second, as for the scores in *Retelling content*, the main effect of candidates’ dialect [F (1, 1) = 0.424, df = 1, *p* = 0.52], the main effect of raters’ dialect [F (1, 1) = 1.817, df = 1, *p* = 0.17], and the interaction between candidates’ dialect and raters’ dialect [F (1, 1) = 0.004, df = 1, *p* = 0.95] were all not statistically significant. Third, with scores in *Pronunciation and intonation* as the dependent variable, the results of ANOVA showed that the main effect of raters’ dialect [F (1, 1) = 0.523, df = 1, *p* = 0.47], the main effect of candidates’ dialect [F (1, 1) = 5.562, df = 1, *p* = 0.02], and the interaction of the candidates’ and raters’ dialect [F (1, 1) = 0.007, df = 1, *p* = 0.93] were all not statistically significant. Last, using scores in *Fluency* as the dependent variable, neither the between-group main effect [F (1, 1) = 1.484, df = 1, *p* = 0.23], the within-group main effect [F (1, 1) = 0.089, df = 1, *p* = 0.77], nor the interaction effect of raters’ dialect × candidates’ dialect [F (1, 1) = 0.053, df = 1, *p* = 0.82] was found statistically significant.

To summarize, the quantitative analysis indicated that neither the main effect of raters’ dialect nor the interaction effect was significant when candidates’ total scores and scores on each dimension were employed as the dependent variable.

### Findings of RQ 2

The current study adopted Winke et al.’s (2013) coding scheme and made some necessary adjustments on coding categories for some new features. Comments were drawn from both rater groups for all eight speech samples. One researcher and the assistant read those comments carefully and coded independently. The coding work was done using QSR NVivo 11.0. The initial intercoder agreement reached approximately 87%. For those incongruences between the coding results, the researcher and the assistant discussed thoroughly. Eventually, a perfect agreement between two coders was achieved. Seven broad themes were identified from analysis of raters’ comments, including (1) candidates’ accent; (2) candidates’ heritage status; (3) raters’ scoring strategy; (4) comments on pronunciation; (5) affect; (6) candidates’ voice; (7) candidates’ intonation.

To elaborate, the first theme was raters’ comments on candidates’ accents, which were further broken down into positive and negative comments. Comments such as “the accent was great” and “it did not impact understanding” were coded as positive. Comments such as “the accent was a bit problematic,” “… made it difficult to score,” and “… left me an awful impression of the candidate” were categorized as negative. Some other references only mentioned the candidates’ accents but without further comment were coded as neutral. Theme two (candidate’s heritage status) was relative to comments of guessing where candidates may come from. The third theme showed raters’ scoring strategy of paying attention to candidates’ pronunciation in the first place. Different from comments on candidates’ accents, raters’ positive or negative comments on candidates’ pronunciation were coded into the fourth coding category. The coding theme of affect related to how rater felt while listening and rating. The ultimate two coding categories were germane to the candidates’ voice and intonation, respectively.

The seven coding themes, the number of raters (including the number of raters from two dialect groups) who made comments associated with the theme, the frequency of references connected to the theme, and the entire numbers of words used in discussing the theme were displayed in Table 3.

**TABLE 3:** Summary of coding themes.

Coding category	Raters	Cantonese-speaking raters	Mandarin-speaking raters	References	Words
1. Candidate’s accent	9	4	5	40	2,394
1.1. Positive	5	2	3	12	571
1.2. Negative	5	2	3	17	1,174
1.3. Neutral	5	3	2	11	649
2. Candidate’s heritage status	7	4	3	14	1,209
3. Rater’s scoring strategy	8	3	5	12	622
4. Comments on pronunciation	7	5	2	20	679
4.1. Positive	7	4	3	14	408
4.2. Negative	4	2	2	6	271
5. Affect	6	3	3	6	223
6. Candidate’s voice	5	1	4	6	256
7. Candidate’s intonation	4	4	0	7	496

The following subsections would probe into three major coding themes relevant to the relationship between raters’ and candidates’ dialects, including (1) candidates’ accent; (2) candidates’ heritage status; (3) raters’ scoring strategy.

### The candidates’ accent

Nine of the fourteen raters reported that they noticed or made further comments on the candidates’ accents while listening and rating. Five raters expressed a positive attitude toward accents. Five raters conveyed negative feelings toward the issue of accent. They commented that accents affected their comprehension of the samples and probably influenced their rating decision. Five raters said that they noticed the accents in the candidates’ speech, but did not comment on this issue.

Two Cantonese-speaking raters and three Mandarin-speaking raters held a positive view of accent. They indicated that having an accent did not matter a lot as long as it did not interfere with understanding, as demonstrated in (1) and (2).

[1] *I noticed that he had a strong accent, the Cantonese accent. But I don’t think it mattered, as long as it did not challenge my understanding* (B8, Cantonese-speaking).

 [2] *When the speaker started to talk, I could easily identify her Mandarin accent. Compared with the last speaker (a Cantonese-accented speaker), I felt more comfortable with her accent* (A9, Mandarin-speaking).

Interestingly, a Cantonese-speaking rater (B6) and her Mandarin-speaking counterpart (A8) seemed to display a feeling of positive bias for familiar accents. They all noted that candidates’ speeches as a whole were not extremely difficult to understand because they were familiar with candidates’ accents. And due to this familiarity, they became tolerant of various difficulties during the rating process, as shown in (3) and (4).

[3] *This speech sample as a whole was slightly difficult to understand. Although I have read the script, I don’t know why he mentioned the words like “garden” and “milk”, so I couldn’t understand what he was saying. But his accent was fine to me, because basically I’m familiar with it* (B6, Cantonese-speaking).

 [4] *The candidate spoke slowly and his pronunciation was not as natural as native speakers. When I listened to this sample, the candidate’s accent reminded me of my English teacher’s speech pattern. Her pronunciation was friendly to me and helped me understand what she was saying* (A8, Mandarin-speaking).

In contrast, two Cantonese-speaking raters and three Mandarin-speaking raters held a negative view on candidates’ accents. Seventeen comments were coded into the negative category. Raters expressed a general concern for candidates’ pronunciation with accents and conveyed dreadful feelings. As illustrated in the following comments, they reported that the annoying accent made them feel uncomfortable, unpleasant and perplexed, leading to unfavorable impressions. They also noted that accents affected their listening process and impeded comprehension. A representative example can be found in the following comment by A3. She stated that the unfamiliar strong accent made it difficult for her to understand the candidate and thus influenced her rating. Ultimately, she only assigned a passing score because of the strong accent.

[5] *In terms of pronunciation, his heavy accent and dialect gave rise to all sorts of difficulties. It can significantly affect my understanding, so he only got a passing mark* (A3, Mandarin-speaking).

Five raters commented that candidates’ pronunciation was not accurate and excellent. They noticed an issue of accents occurring in candidates’ performances. However, the Cantonese-speaking raters were more likely than the Mandarin-speaking raters to recognize the Cantonese accent and Mandarin-speaking raters were better at identifying Mandarin accent. A pair of examples can be found in (6) and (7).

[6] *What I hear is that he had a Cantonese accent regarding pronunciation. One thing by the way, I think most speech samples have rhymes. I felt that the feature of rhymes at the end of every sentence or word was like a Cantonese accent* (B7, Cantonese-speaking).

 [7] *The speaker’s Mandarin accent was not native-like, because his pronunciation was not very good, the intonation was basically flat, and his pronunciation seemed to have a strong accent, and some words were not accurately pronounced* (A3, Mandarin-speaking).

### The candidates’ heritage status

Seven raters reported wondering about the candidates’ language of origin and guessing where they came from. Fourteen comments were coded into this category.

In the following excerpts, one Cantonese-speaking and two Mandarin-speaking raters discussed how they recognized accents. B1 claimed that the Mandarin candidate’s accent was identical to one of his friends who did not live in the Cantonese speaking area.

[8] *I can perceive that her English accent was totally different from the English spoken by the native people of Guangdong province. Her accent was very close to one of my friends, but she did not belong to our ethnic group* (B1, Cantonese-speaking).

A1 mentioned that the typical phonetic error of mixing /n/ with /l/ reminded her of Southern accent, as shown in (9). Her prior experience with individuals who spoke with strong Southern accent also made her identify the candidate’s Southern accent.

[9] *This person had a strong and obvious Southern accent, which had an effect on his pronunciation. For example, it was related to the common pronunciation mistake in South China that mixed /n/ with /l/. A certain phrase did give me a deep impression, I remember it was “there was no answer”, in which /n/ is mispronounced as /l/ by him. Anyway, it was possible that the Southern accent had a certain influence on pronunciation* (A1, Mandarin-speaking).

Besides, A6 inferred that a candidate might be from South China from the way how she pronounced.

[10] *My first thought is that this student’s accent suggested that she might be from the South, as her English pronunciation was a little strange, that is, she couldn’t pronounce each sound correctly. It seemed that she only used the front part of the tongue, and seldom the back part* (A6, Mandarin-speaking).

What is interesting about several Mandarin-speaking raters who were unfamiliar with Cantonese is that they took some Cantonese candidates for Indian, Thai, black American English speakers, as shown in (11), which was in accordance with Ballard and Winke’s (2017) finding that non-native speakers of English always feel difficult to ascertain the origin of an accent.

[11] *When I listened to it for the first and second time, I thought that the accent of this person was very similar to Thai English. You know, it was really difficult to understand, and it was kind of weird* (A2, Mandarin-speaking).

However, unlike the Mandarin-speaking raters who were unfamiliar with the Cantonese, four Cantonese-speaking raters succeeded in identifying candidates’ heritage status, as demonstrated in the following comments. They indicated that notable features in candidates’ pronunciation enabled them to determine that the candidates might come from Guangdong province. This can be seen in the example of rater B5. She made a speculation in (12) about where the candidate might come from and confirmed that the candidate was a Cantonese in a short time based on the accented pronunciation of Cantonese.

[12] *After listening for just 10 s, I could tell that this student must be a Cantonese, because his pronunciation sounded odd, which only exists in Cantonese people. For some words, the /r/ was going to be a little bit skewed toward /l/. For instance, they pronounce “very” as “vely”. I think these are typical features of Cantonese pronunciation, so I probably listened to it for the first 10 s or so and knew he was a Cantonese* (B5, Cantonese-speaking).

### Raters’ scoring strategy

Among the raters who participated in the stimulated recall session, half indicated using the strategy of prioritizing pronunciation while scoring. They expressed the belief that if the candidate’s pronunciation was accurate and excellent at first, it would leave a pleasant impression on them, hence they would assign a higher score. It appeared that candidates’ performance in *pronunciation* had a significant effect on rating, as illustrated in (13) and (14).

[13] *According to the four dimensions of the rating scale, first of all, I would assess whether his pronunciation is good and accurate as soon as he opens his mouth. I think there was an evaluation standard in my mind* (A7, Mandarin-speaking).

 [14] *First of all, if he speaks out, his pronunciation is very good, the first impression will be good, then if his intonation is good and smooth, and I will definitely give him a high score* (B8, Cantonese-speaking).

## Discussion

### Discussion of RQ 1

The quantitative results demonstrated that there were no appreciable differences in the consistency of each rater when judging test performance, which was in agreement with the findings of other studies that acceptable consistency was obtained in the ratings of raters no matter whether they were familiar or unfamiliar with the first language of the speaker being assessed (Xi and Mollaun, 2011; Winke et al., 2013; Park, 2020). From a theoretical perspective, the findings of this study offer evidence against existence of a shared-dialect effect in rating candidates’ performance on the retelling task and bridge the gap of empirical study on the shared-L1 effect within assessment context, especially in the Chinese context which has thus far been the focus of little research.

The reasonably high scoring consistency of the two rater groups might be attributed to the training that both rater groups received. All raters were required to participate in the training session, which resulted in their greater understanding of the rationale for each score. Rater training was effective in helping raters to gain consensual understanding of the categories and criteria represented in the rating rubric and to adopt a common frame of reference (Saito, 2008), leading to greater improvement in the level of agreement between raters. Furthermore, rater reliability reflected *via* scores is not necessarily the sole indicator of an accredited rater’ assessment literacy. In this research, in order to guarantee the validity of the ratings, the training calibration test standards incorporated expert raters’ reasons for ratings into the measurement of their understanding of the rating scale. As a result, the integration of both psychometric approach and hermeneutic approach (Petruzzi, 2008) to rater training substantially improved the consistency and interpretability of ratings. It may not be surprising that raters might be guided by their experience in the rating process in the absence of rater training, and they tended to determine scores differently based on different levels of experience using the language being tested (Winke and Gass, 2013). Overall, rater training seemed to have helped raters score consistently and confidently.

The two-factor ANOVA analysis found no statistically significant interactions between raters’ dialect and candidates’ dialect in the total score and in each rating category, suggesting that the two rater groups were equivalent in the scores assigned to the two candidate groups. These results rejected the hypothesis that raters who share the same dialect with the candidate would give a higher score to that candidate than those who do not. Neither the Mandarin-speaking raters nor the Cantonese-speaking raters showed a shared-dialect effect. These findings differed from some published studies (Harding, 2012; Dai and Roever, 2019), but they were aligned with previous studies showing inconsistent effects based on a shared language background (Abeywickrama, 2013; Crowther et al., 2016).

Aside from the aforementioned rater training, the null result could be explained by the analytic rating scale employed in the present study. Previous studies have found that both rating criteria and rater training could become a crucial factor in raters’ rating outcomes (Xi and Mollaun, 2011). Typically, raters engage in impressionistic judgment when applying a holistic rubric to rate test-takers’ overall speech quality (Xi and Mollaun, 2009). Since the present study used an analytic rubric, raters had to adjust their typical rating behavior and resort to more analytic evaluations in judging the speaking proficiency of examinees who shared the same dialect with them, which may have helped them engage in more reliable and valid evaluations. Moreover, the benchmark samples as exemplars of each score level of the rating scale could guide the raters to determine how similar a sample was to the exemplar, which enhanced raters’ understanding of descriptions at different score levels. Hence, raters could articulate scores in some way consistent with the rating scale and provide more accurate and consistent assessments.

The findings tend to suggest that both groups of raters were capable of rating reliably and consistently. Evaluations of oral performance by the two rater groups resulted in the same or roughly similar outcome in terms of aggregate scores. While the selection of raters for the current study cannot be deemed to represent the broader population, these findings provide sound grounds for including both Cantonese-speaking raters and Mandarin-speaking raters in assessing speaking ability in the CELST. It seems that the language background of raters may not matter for scoring purposes in a testing context, and raters from different language backgrounds can be employed interchangeably as long as they have been sufficiently trained.

### Discussion of RQ 2

The second research question is whether raters are aware that sharing the same dialect with the candidates might impact their rating process. Results from the qualitative analysis indicated that most raters could recognize the candidates’ native dialect by their accents. On the whole, accent had little effect on their understanding. It appeared that Cantonese-speaking raters were more capable of identifying candidates’ Cantonese accent, compared with their Mandarin-speaking counterparts.

Some raters indicated that awareness of accents and their familiarity with that accent played a role in the comprehension, and potentially affected their scoring process. The present findings seem to be consistent with previous research which showed that scores were affected by accent familiarity, resulting in higher scores (Winke and Gass, 2013; Winke et al., 2013). For example, the Cantonese-speaking rater (B6) displayed a feeling of positive bias in rating candidates with familiar accent. She commented that candidates’ speech as a whole was not extremely difficult to understand because she was familiar with this accent. There was, therefore, a possibility for her to become tolerant of accented pronunciation.

Although the two rater groups did not significantly differ in numerical ratings of candidates’ oral performance, some raters participated in the stimulated recall stage reported that familiarity with candidates’ accents potentially affected their rating decisions. This finding was aligned with the results of prior studies showing discrepancies between raters’ assigned ratings and self-perceptions (Xi and Mollaun, 2011; Huang, 2013).

These mismatching results might be due to the mediating effect of raters’ attitudes as suggested by Huang (2013). In the present study, Mandarin-speaking raters shared similar attitudes toward the Cantonese accent with the Cantonese rater group. The lack of significant difference in numerical rating may therefore be explained by the similar attitude between the two rater groups. Additionally, the present study focused on the retelling task, which is an integrated task rather than a task that lay mere emphasis on the pronunciation. The core of scoring integrated tasks is the overall oral proficiency rather than pronunciation, which might affect raters’ scoring decision. It is possible that a shared-dialect effect is more of a concern with tasks that focuses on pronunciation, like reading-aloud, than with tasks that assess comprehensive speaking ability. Previous studies investigating a possible shared-L1 effect in listening tests suggested that a shared-L1 effect seems to exert different impact on various task types (Dai and Roever, 2019). As a result, the role of task type deserves further exploration.

The potential for test bias in English oral assessment featuring raters with regional dialects has been proved from the cognitive perspective. It provides a foundation for further research on the effect of regional dialects in oral tests, and suggests that a shared-dialect effect is more likely to occur. Although such effect may be made ‘steerable’ *via* rigorous training, the conflicting results still raise a cautionary red flag that raters’ bias caused by personal dialects requires careful monitoring.

## Conclusion

In summary, the shared-dialect benefit was neither observed with Mandarin-speaking raters nor with Cantonese-speaking raters, despite that some raters attended to candidates’ accent/dialect and indicated that awareness of accents and their familiarity with the accents affected their comprehension of the speech samples and potentially influenced their scoring process. The above findings add to our knowledge of the shared-dialect benefit and support the claim that including both group raters in the CELST is valid on the condition that rigorous rater training has been provided.

The current study is not without limitations. First, the validity of the whole research procedure could be improved if it was done under normal circumstances. The outbreak of the *COVID-19* pandemic definitely reduced the effectiveness and efficiency of the training procedure and the stimulated recall method. Second, the current study only examined differences in rating behavior among raters at the group level through the classical statistical analyses, which may not be powerful enough to detect differences at the individual level. It might be the case that a higher score assigned by one rater to the candidate was offset by a lower score awarded by another rater in the group, and these variations were not captured in the current analysis treating raters as a group (Huang et al., 2016). As such, more sophisticated statistical analyses such as the Multi-Faceted Rasch model should be employed in future to gain more fine-grained insights into the rater variability. Third, there is a small chance that rating decisions were affected by accent familiarity, but the effect did not entirely demonstrate in the present study. In particular, the mismatch between raters’ assigned ratings and self-perceptions demands closer examination of raters’ decision-making process. More qualitative data should be collected through other methods (like interview) to triangulate the findings. Finally, since the raters in this study were all young inexperienced female postgraduates, future studies could employ male and/or experienced raters to improve generalizability of the present findings.

## Data availability statement

The raw data supporting the conclusions of this article will be made available by the authors, without undue reservation.

## Ethics statement

The studies involving human participants were reviewed and approved by the Ethics Committee at School of Foreign Languages, South China University of Technology. The patients/participants provided their written informed consent to participate in this study.

## Author contributions

YX: conceptualization, organization, and revision. MH and JC: revision and expansion. YZ: writing—original draft. All authors contributed to the article and approved the submitted version.

## References

[B1] Abeywickrama P. (2013). Why not non-native varieties of English as listening comprehension test input? *RELC. J*. 44 59–74. 10.1177/0033688212473270

[B2] Bachman L. F. Palmer A. S. (1996). *Language testing in practice.* Oxford: Oxford University Press.

[B3] Ballard L. Winke P. (2017). “Students’ attitude towards English teachers’ accent: The interplay of accent familiarity, comprehensibility, intelligibility, perceived native speaker status, and acceptability as a teacher,” in *Second language pronunciation assessment: Interdisciplinary perspectives*, eds Isaacs T. Trofimovich P. (Bristol: Multilingual Matters), 121–140. 10.21832/ISAACS6848

[B4] Barkana B. D. Patel A. (2020). Analysis of vowel production in Mandarin/Hindi/American-accented English for accent recognition systems. *Appl. Acoust.* 162:107203. 10.1016/j.apacoust.2019.107203

[B5] Bent T. Bradlow A. R. (2003). The interlanguage speech intelligibility benefit. *J. Acoust. Soc. Am*. 114 1600–1610. 10.1121/1.1603234 14514213

[B6] Bradlow A. Bent T. (2008). Perceptual adaptation to non-native speech. *Cognition* 106 707–729. 10.1016/j.cognition.2007.04.005 17532315 PMC2213510

[B7] Canagarajah S. (2006). Changing communicative needs, revised assessment objectives: Testing English as an international language. *Lang. Assess. Q.* 3 229–242. 10.1207/s15434311laq0303_1

[B8] Carey M. D. Mannel R. H. Dunn P. K. (2011). Does a rater’s familiarity with a candidate’s pronunciation affect the rating in oral proficiency interviews? *Lang. Test*. 28 201–219. 10.1177/0265532210393704

[B9] Cargile A. C. (1997). Attitudes toward Chinese-accented speech: An investigation in two contexts. *J. Lang. Soc. Psychol*. 16 434–443. 10.1177/0261927X970164004

[B10] Crowther D. Trofimovich P. Isaacs T. (2016). Linguistic dimensions of second language accent and comprehensibility: Nonnative listeners’ perspectives. *J. Second Lang. Pronunciation* 2 160–182. 10.1075/jslp.2.2.02cro 33486653

[B11] Dai D. W. Roever C. (2019). Including L2-English varieties in listening tests for adolescent ESL learners: L1 effects and learner perceptions. *Lang. Assess. Q.* 16 64–86. 10.1080/15434303.2019.1601198

[B12] Davis L. (2015). The influence of training and experience on rater performance in scoring spoken language. *Lang. Test*. 33 117–135. 10.1177/0265532215582282

[B13] Elder C. Barkhuizen G. Knoch U. von Randow J. (2007). Evaluating rater responses to an online training program for L2 writing assessment. *Lang. Test.* 24 37–64. 10.1177/0265532207071511

[B14] Frost K. Elder C. Wigglesworth G. (2012). Investigating the validity of an integrated listening-speaking task: A discourse-based analysis of test takers’ oral performances. *Lang. Test.* 29 345–369. 10.1177/0265532211424479

[B15] Gass S. M. Mackey A. (2000). *Stimulated recall methodology in second language research.* New Jersey, NJ: Lawrence Erlbaum Associates.

[B16] Gass S. M. Varonis E. M. (1984). The effect of familiarity on the comprehensibility of nonnative speech. *Lang. Learn*. 34 65–87. 10.1111/J.1467-1770.1984.TB00996.X

[B17] Green A. (1998). *Verbal protocol analysis in language testing research: A handbook.* Cambridge: Cambridge University Press.

[B18] Gui M. (2012). Exploring differences between Chinese and American EFL teachers’ evaluations of speech performance. *Lang. Assess. Q.* 9 186–203. 10.1080/15434303.2011.614030

[B19] Han W. Brebner C. M. Mcallister S. (2015). Redefining ‘Chinese’ L1 in SLP: Considerations for the assessment of Chinese bilingual/bidialectal language skills. *Int. J. Speech Lang. Pathol*. 18 135–146. 10.3109/17549507.2015.1081285 27172849

[B20] Harding L. (2012). Accent, listening assessment and the potential for a shared-L1 advantage: A DIF perspective. *Lang. Test.* 29 163–180. 10.1177/0265532211421161

[B21] Hayes-Harb R. Smith B. L. Bent T. Bradlow A. R. (2008). The interlanguage speech intelligibility benefit for native speakers of Mandarin: Production and perception of English word-final voicing contrasts. *J. Phon.* 36 664–679. 10.1016/j.wocn.2008.04.002 19606271 PMC2709866

[B22] Hoskens M. Wilson M. (2001). Real-time feedback on rater drift in constructed-response items: An example from the golden state examination. *J. Educ. Meas*. 38 121–145. 10.1111/j.1745-3984.2001.tb01119.x

[B23] Huang B. H. (2013). The effects of accent familiarity and language teaching experience on raters’ judgments of non-native speech. *System* 41 770–785. 10.1016/j.system.2013.07.009

[B24] Huang B. Alegre A. Eisenberg A. (2016). A cross-linguistic investigation of the effect of raters’ accent familiarity on speaking assessment. *Lang. Assess. Q.* 13 25–41. 10.1080/15434303.2015.1134540

[B25] Isaacs T. (2016). “Assessing speaking,” in *Handbook of second language assessment*, eds Tsagari D. Banerjee J. (Berlin: De Gruyter Mouton), 131–146. 10.1515/9781614513827-011

[B26] Isaacs T. Thompson R. I. (2013). Rater experience, rating scale length, and judgments of L2 pronunciation: Revisiting research conventions. *Lang. Assess. Q.* 10 135–159. 10.1080/15434303.2013.769545

[B27] Jin Y. Fan J. (2011). Test for English majors (TEM) in China. *Lang. Test.* 28 589–596. 10.1177/0265532211414852

[B28] Kang O. Rubin D. Kermad A. (2019a). The effects of training and rater differences on oral proficiency assessment. *Lang. Test*. 36 481–504. 10.1177/0265532219849522

[B29] Kang O. Thomson R. Moran M. (2019b). The effects of international accents and shared first language on listening comprehension tests. *TESOL Q.* 53 56–81. 10.1002/tesq.463

[B30] Kim H. J. (2015). A qualitative analysis of rater behavior on an L2 speaking assessment. *Lang. Assess. Q.* 12 239–261. 10.1080/15434303.2015.1049353

[B31] Law N. C. So L. K. (2006). The relationship of phonological development and language dominance in bilingual Cantonese-Putonghua children. *Int. J. Biling*. 10 405–427. 10.1177/13670069060100040201

[B32] Lee Y. S. Vakoch D. A. Wurm L. H. (1996). Tone perception in Cantonese and Mandarin: A cross-linguistic comparison. *J. Psycholinguist. Res*. 25 527–542. 10.1007/BF01758181 8865624

[B33] Li R. (1989). Classification/distribution of Chinese dialects. *Dialect* 4 241–259.

[B34] Lindemann S. (2005). Who speaks “broken English”? US undergraduates’ perceptions of non-native English. *Int. J. Appl. Linguist*. 15 187–212. 10.1111/j.1473-4192.2005.00087.x

[B35] Lindemann S. (2017). “Variation or ‘error’? Perception of pronunciation variation and implications for assessment,” in *Second language pronunciation assessment: Interdisciplinary perspectives*, eds Isaacs T. Trofimovich P. (Bristol: Multilingual Matters), 193–209. 10.21832/9781783096855-013

[B36] Liu M. (2013). Validation of test of English major band 4 (TEM4) story retelling oral test rating scale. *J. Zhejiang Univ.* 43 187–194.

[B37] Major R. C. Fitzmaurice S. F. Bunta F. Balasubramanian C. (2002). The effects of nonnative accents on listening comprehension: Implications for ESL assessment. *TESOL Q.* 36 173–190. 10.2307/3588329

[B38] McNamara T. F. (1996). *Measuring second language performance.* Harlow: Longman.

[B39] Munro M. J. Derwing T. M. Morton S. L. (2006). The mutual intelligibility of L2 speech. *Stud. Second Lang. Acquis*. 28 111–131. 10.1017/S0272263106060049

[B40] National Advisory Committee for Foreign Language Teaching (2004). *Syllabus for test for English majors (Grade 8).* Shanghai: Shanghai Foreign Language Education Press.

[B41] Ockey G. J. French R. (2016). From one to multiple accents on a test of L2 listening comprehension. *Appl. Linguist.* 37 693–715. 10.1093/applin/amu060

[B42] Park M. S. (2020). Rater effects on L2 oral assessment: Focusing on accent familiarity of L2 teachers. *Lang. Assess. Q.* 17 231–243. 10.1080/15434303.2020.1731752

[B43] Petruzzi A. (2008). Articulating a hermeneutic theory of writing assessment. *Assess Writ.* 13 219–242. 10.1016/j.asw.2008.10.005

[B44] Saito H. (2008). EFL classroom peer assessment: Training effects on rating and commenting. *Lang. Test.* 25 553–581. 10.1177/0265532208094276

[B45] Stevenage S. V. Clarke G. McNeill A. (2012). The “other-accent” effect in voice recognition. *J. Cogn. Psychol*. 24 647–653. 10.1080/20445911.2012.675321

[B46] Stibbard R. M. Lee J. (2006). Evidence against the mismatched interlanguage speech intelligibility benefit hypothesis. *J. Acoust. Soc. Am*. 120 433–442. 10.1121/1.2203595 16875239

[B47] Tauroza S. Luk J. C. (1997). Accent and second language listening comprehension. *RELC. J*. 28 54–71. 10.1177/003368829702800104

[B48] Thomas D. R. (2006). A general inductive approach for analyzing qualitative evaluation data. *Am. J. Eval.* 27 237–246. 10.1177/1098214005283748

[B49] Wei J. Llosa L. (2015). Investigating differences between American and Indian raters in assessing TOEFL iBT speaking tasks. *Lang. Assess. Q.* 12 283–304. 10.1080/15434303.2015.1037446

[B50] Weigle S. C. (1998). Using FACETS to model rater training effects. *Lang. Test.* 15 263–287. 10.1177/026553229801500205

[B51] Winke P. (2012). “Rating oral language,” in *The encyclopedia of applied linguistics*, ed. Chapelle C. A. (New York, NY: Blackwell Publishing Ltd), 1–8. 10.1002/9781405198431.wbeal0993

[B52] Winke P. Gass S. (2013). The influence of second language experience and accent familiarity on oral proficiency rating: A qualitative investigation. *TESOL Q.* 47 762–789. 10.1002/tesq.73

[B53] Winke P. Gass S. Myford C. (2013). Raters’ L2 background as a potential source of bias in rating oral performance. *Lang. Test.* 30 231–252. 10.1177/0265532212456968

[B54] Xi X. Mollaun P. (2009). *How do raters from India perform in scoring the TOEFL iBT*™* Speaking Section and what kind of training helps?* (TOEFL iBT Research Report RR-09-31). Princeton, NJ: Educational Testing Service.

[B55] Xi X. Mollaun P. (2011). Using raters from India to score a large-scale speaking test. *Lang. Learn.* 61 1222–1255. 10.1111/j.1467-9922.2011.00667.x

[B56] Xie X. Fowler C. A. (2013). Listening with a foreign-accent: The interlanguage speech intelligibility benefit in Mandarin speakers of English. *J. Phon*. 41 369–378. 10.1016/j.wocn.2013.06.003 24293741 PMC3842167

[B57] Xing Q. Wu X. Wang J. Zhang Z. (2021). The influence of the matching of modality presentation mode and perceptual learning style on the bidialectal switching cost of Cantonese-Mandarin. *Acta Psychol. Sin.* 53 1059–1070. 10.3724/sp.j.1041.2021.01059

[B58] Xu Y. Zeng Y. (2015). How to deal with students’ pronunciation errors — A study based on the corpus of test-takers’ performance in the Computer-based English listening and speaking test (CELST) of Gaokao (Guangdong Version). *Curric. Teach. Mater. Method* 35 90–96.

[B59] Yang Y. Chen S. Chen X. (2020). “F0 patterns in Mandarin statements of Mandarin and Cantonese speakers,” in *Proceedings of the annual conference of the international speech communication association, INTERSPEECH*, (Shanghai: The Hong Kong Polytechnic University), 4163–4167. 10.21437/interspeech.2020-2549

[B60] Zhang B. Elder C. (2011). Judgments of oral proficiency by non-native and native English speaking teacher raters: Competing or complementary constructs? *Lang. Test.* 28 31–50. 10.1177/0265532209360671

[B61] Zhang F. (2014). Washback of the listening and speaking test in NMET Guangdong: Teachers’ BAK cognition system and test preparation behaviors. *Foreign Lang. Test. Teach.* 3 44–49.

[B62] Zou S. Xu Q. (2016). A washback study of the test for English majors for grade eight (TEM8) in China — From the perspective of university program administrators. *Lang. Assess. Q.* 14 140–159. 10.1080/15434303.2016.1235170

