# Supplementary material for: Investigating a shared-dialect effect between raters and candidates in English speaking tests
Source: Front Psychol. 2023 Mar 30;14:1143031. doi: 10.3389/fpsyg.2023.1143031 (PMC10099360; doi:10.3389/fpsyg.2023.1143031)
Supplement: Supplementary file 1 [file Data_Sheet_1.doc]

**Appendix A**

**The script of Story Retelling task in the CELST in Guangdong NMET, 2013**

The Postman

Tom was a postman. Every time he delivered a letter to Mrs. Brown, he had to finish his work quite late because she lived far away. However, Tom did not mind this, for Mrs. Brown always kindly invited him in for a drink.

When Tom entered the gate of Mrs. Brown’s house yesterday, he didn’t see her working in the garden. He was surprised, because she usually spent afternoons there when the weather was fine. Tom thought she might be in the kitchen. Then he went to the back of the house. But the door was locked. So he returned to the front of the house and knocked hard on the door. There was no answer. Tom thought that this was very strange because he knew Mrs. Brown seldom left the house.

Just then he noticed that her milk, delivered early in the morning, was still at the door. If Mrs. Brown had not taken in her milk, perhaps she was ill. Tom walked around the house and found an open window. He went inside and found Mrs. Brown was lying on the floor of the living room. Tom realized that there was little he could do. He called for an ambulance.

Part C: Retelling

*In this part, you’re required to listen to a monologue twice, and then retell it based on what you have heard in your own words.*

**Outline:** There was no answer when Tom delivered a letter to Mrs. Brown, so he came in through the open window and saw her lying on the floor.

**Keywords:** postman; deliver a letter; house; milk; lie

(Reproduced with permission from the Education Examinations Authority of Guangdong Province (EEAGP), the developer of CELST.)

**Appendix B**

**Listener Judge Strength and Identifiability of Accent Scale**

Listen to each recording clip:

1. **Decide the accentedness**

**Band 1**
The speaker’s accent is very similar to what I am used to.

I can concentrate on listening without any problem.

I can easily understand the recording.

**Band 2**
The speaker’s accent is slightly different from what I am used to.

I can concentrate on listening without too much trouble.

I can understand the recording to a large extent.

**Band 3**
The speaker’s accent is different from what I am used to.

I find it slightly challenging to concentrate on listening.

I can roughly understand the recording.

**Band4**
The speaker’s accent is very different from what I am used to.

I need to concentrate on listening more than usual.

I have limited understanding of the recording.

**Band 5**
The speaker’s accent is noticeably different from what I am used to.

I have to excessively concentrate on listening.

I can barely understand the recording.

1. **Identify the accent**
2. Cantonese B. Mandarin C. not know

**Appendix C**

**Rater Background Questionnaire**

1. Age: ________

2. Gender: Male________ Female________

3. University: ________

4. Major: ________

5. Grade of postgraduate study: ________

6. Dialect/Mother tongue (primary language spoken in your childhood home): ________

7. Have you had any hearing or speech impairment? Yes________ No________

If yes, please explain: ______________

8. For how long have you studied English? ________Years

When did you start to study English? _______

Where did you start to study English? ________

9. Which language other than English have you studied? ________

10. Have you been to foreign countries for study before?

Yes________ No________

If yes, where: ________

11. What is your English level?

TEM4____ TEM8_____ Other (please specify) ________

12. How would you best describe your familiarity with Cantonese?

(A) Strongly unfamiliar. (B) Unfamiliar. (C) Neutral. (D) Familiar. (E) Strongly familiar.

13. How would you best describe your attitude towards Cantonese?

(A) Strongly dislike. (B) Dislike. (C) Neutral. (D) Like. (E) Strongly like.

14. Have you scored any oral English tests before? Yes________ No________

If yes, how many times: ________

**Appendix D**

**The rating scale of the Story Retelling task in the CELST in Guangdong NMET**

|  | **Content score** | | **Holistic score** | |
| --- | --- | --- | --- | --- |
| Scoring method | To score according to the accuracy of information unit, 1.5 mark for each. (10 information units in total.) | | To score according to the content, language, fluency and pronunciation. | |
| Scale | Mark | Criteria | Mark | Criteria |
| A | 1.5 | Completely correct | 7-9 | ·Be faithful to the original content.  ·Appropriate use of language, conformity with the norm.  ·Fluency in expression.  ·Pronunciation and intonation errors do not impede understanding. |
| B | 0.5-1.0 | Basically correct | 4-6 | ·Basically cover the main idea of the story.  ·Fairly appropriate use of language, and basic conformity with the norm. A few errors may exist, yet don’t impede understanding.  ·Fairly fluent, yet some short pauses may exist in retelling.  ·Some errors about pronunciation and/or intonation may appear, but they do not impede understanding. |
| C | 0 | Completely wrong | 0-3 | ·Fail to cover the main idea of the story.  ·Poor language appropriateness and a number of language errors.  ·Not fluent.  ·Pronunciation and intonation errors impede understanding. |
| A note of information unit | ·Scoring should be done with an emphasis on information. As long as the information unit is covered, then a mark should be awarded.  ·Language errors are considered in the holistic score. | | | |

(Reproduced with permission from the Education Examinations Authority of Guangdong Province (EEAGP), the developer of CELST.)

**Appendix E**

**Rating criteria (Liu, 2013)**

| **Scale** | **Mark** | **Grammar, vocabulary and expression** | **Retelling content** | **Pronunciation and intonation** | **Fluency** |
| --- | --- | --- | --- | --- | --- |
| Excellent | 4 | Almost no grammatical mistakes; appropriate use of vocabulary and accurate expression of ideas; complete sentence structures and coherent expressions; a wide range of vocabulary and sentence patterns; strong ability to use language. | Able to understand the story completely; retelling content is consistent with the original story and details are accurate; inclusion of complete relevant information; systematic development and strong logic between ideas. | Accurate pronunciation and clear speech; natural intonation; leave a good impression on the audience. | Fluent and well-paced speech; with occasional appropriate adjustments; fluid expression. |
| Good | 3 | A small amount of errors in grammar, vocabulary and expression of ideas; fairly complete sentence structures and coherent expressions; a fairly wide range of vocabulary and sentence patterns;  fairly strong ability to use language. | Able to understand the story; retelling content is basically consistent with the original story and details are fairly accurate; inclusion of most relevant information;  fairly systematic development and strong logic between ideas. | Basically accurate pronunciation and clear speech; basically natural intonation; can hardly impede understanding. | May include occasional hesitations; able to make timely modifications and adjustments; fairly fluid expression. |
| Pass | 2 | A fairly large amount of errors in grammar, vocabulary and expression of ideas;  fairly incomplete sentence structures and incoherent expressions; a fairly limited range of vocabulary and sentence patterns;  fairly weak ability to use language. | Able to basically understand the story; able to basically retell the main content of the story but miss few important information; lack systematic development and logic between ideas; unable to reorganize the original story smoothly. | Some inaccurate pronunciations and unnatural intonation patterns; can easily impede understanding. | Speech is markedly disfluent due to frequent hesitations and pauses; have some modifications and adjustments;  speak either too fast or too slowly; require listeners’ effort to understand. |
| Fail | 1 | A large amount of serious errors in grammar, vocabulary and expression of ideas; incomplete and incoherent sentences; a limited range of vocabulary and sentence patterns;  basically unable to retell the story. | Unable to understand the story; a serious mismatch between the retold story and the original version; miss key important information; confusing logic between ideas; unable to retell the story. | Inaccurate pronunciation and unclear speech; unnatural intonation; unable to express information effectively. | The speech is extremely disfluent due to frequent hesitations and pauses; with constant repetitions and modifications; painstakingly difficult to understand. |

(Reproduced with permission from Doctor Liu (the author of the article in which the rating criteria is extracted) and the Journal of Zhejiang University (Humanities and Social Sciences Edition) (the publisher of the article).)

Liu, M. (2013). Validation of Test of English Major Band 4 (TEM4) story retelling oral test rating scale. *Journal of Zhejiang University (Humanities and Social Sciences Edition)*, 43(6), 187-194.
